# *Channa striata *(Ikan Gabus) Extract and the Acceleration of Tuberculosis Treatment: A True Experimental Study

**DOI:** 10.1155/2019/8013959

**Published:** 2019-03-19

**Authors:** Isa Ma'rufi, Khaidar Ali, Ign Arya Sedemen, P. Purwanto, Abu Khoiri

**Affiliations:** ^1^Department of Environmental Health and Occupational Safety, School of Public Health, University of Jember 68121, Indonesia; ^2^Jember Chest Hospital, 68118 East Java, Indonesia

## Abstract

Tuberculosis is international health problem, which is classified in Global Emergency disease since 1992. The objective of the study is to determine the effect of* Channa striata* extract toward the acceleration of tuberculosis treatment. The study used true experiment, in which the intervention of the study was* Channa striata *supplementation to respondent. In addition, Chi-square was used to analyze the data with SPSS version 22. The result is the proportion of respondent classified in negative category in intervention group within week 0, week 1, week 2, week 3, and week 4 being 10.7%, 35.9%, 56.3%, 70.9%, and 90.3%, respectively. Besides, the proportion of respondent classified in negative category in control group within week 0, week 1, week 2, week 3, and week 4 was 13.4%, 23.7%, 37.1%, 49.5%, and 68%, respectively. Based on Chi-square test, the P value of* Channa striata *supplementation toward the acceleration of tuberculosis treatment week 1, week 3, and week 4 is 0.045, 0.019, and 0.005 (P< *α*; *α*=0.05), respectively. It means that there were differences between* Channa striata *supplementation and acceleration of tuberculosis treatment among respondent. Therefore,* Channa striata* treatment was significantly related to the acceleration of tuberculosis recovery.

## 1. Introduction

Tuberculosis (TB) is an international health problem, which is classified as Global Emergency Disease since 1992. Based on WHO report 2004, there are 8.8 million of new tuberculosis cases in 2002 [1], and one-third of world population has been infected by* Mycobacterium tuberculosis* [2]. Global Tuberculosis Report 2017 noted that 10.4 million people (90% adults; 65% male; 10% people living with HIV) suffered TB and caused 1.7 million people dead in 2016 [3]. In 2016, most of tuberculosis cases occur in South-East Asia (45%), in which 1,020 cases are found in every 261,000 populations [3]. Therefore, tuberculosis becomes important health issue worldwide.

Tuberculosis is deadly disease worldwide [4], which caused 34.5 dead per 100,000 populations on low-income countries in 2015 [5]. In Indonesia, tuberculosis is the main occasion of death in infectious disease [6]. According to Global Tuberculosis Report 2017, Indonesia has the highest incident of tuberculosis in worldwide after India [3] whose rank elevated from 2014 [7]. East Java is one of provinces in Indonesia that has high number of tuberculosis cases with 41,404 cases. Furthermore, Surabaya that is one of the biggest cities in East Java has contributed with the high tuberculosis incidents followed by Jember and Banyuwangi with 3,990 cases, 3,334 cases, and 1,760 cases, respectively. In 2011, the incident of tuberculosis in Jember is reported in 2,182 cases, which is increased from 2010 with 1,963 cases.

The risk factors of the development of tuberculosis are (1) the risk of* Mycobacterium tuberculosis* infection and (2) the risk of the progressivity of tuberculosis infection [8, 9]. Those risk factors have correlation toward the deficiency of macro- and micronutrient [9, 10]. Furthermore, the vulnerable individual of tuberculosis suffers malnutrition [11] toward immunodeficiency mechanism [12]. Besides, tuberculosis is decreasing the body mass and micronutrient deficiency through increasing the energy-need, changing the metabolic process, and decreasing the appetite level [11].

The nutrient supplement can improve the recovery of tuberculosis patient [12]. The micronutrient supplements that had been investigated related to tuberculosis treatment are zinc, arginine, selenium, iron, copper, vitamins A, C, D, and E, and their combination [11, 12]. Paton describes that the effect of macronutrient (high-energy supplements; protein 6.25 g; carbohydrate 20.2 g; fatty 4.92 f; 150 kkal/100 mL; Ensure Plus; Abbott Laboratories, Columbus, OH) toward the increasing of body mass of tuberculosis patient is significantly different from control group (2.57 ± 1.78 compared with 0.84 ± 0.89 kg; P= 0.001) in Singapore [13]. The result of that study is confirmed positively by a systematic review on The Cochrane Library that is conducted by Abba [11]. Therefore, it can be reference as Evidence-Based Medicine (EBM).

*Channa striata *(Ikan Gabus) is one of essential fatty acid and essential amino acid (protein) sources that is cheap and comprehensive. The extract of* Channa striata *contains 16 kinds of amino acids and 8 kinds of essential amino acid such as arginine, threonine, valine, methionine, isoleucine, leucine, phenylalanine, and lysine [14]. Furthermore, the extract of* Channa striata *has 8 kinds of fatty acid and two kinds of essential fatty acid that is classified in omega-6 group such as linoleate acid (C18:2) and arachidonic acid (C20:4) [14].

The study of* Channa striata *supplement related to clinical aspect has been conducted twice in Indonesia. The first research that was conducted among patients in Wahidin Sudirohusodo Hospital was written by Nurpudji Astuti [15]. The result of this study is not published on both national and international events; however the result has already registered to authorize as patent product. The patent number is P00200600144 and published on 8 March 2009 by Ministry of Justice. The second research was simply conducted with quasiexperimental method toward 14 chronic pulmonary respiratory disease patients (n control= 7, n intervention=7) in RS. Paru Jember [16].

The objective of the study is to determine the effect of* Channa striata *(Ikan Gabus) extract toward the acceleration of TB patient treatment, in which the acceleration of tuberculosis treatment in this study is shortening the tuberculosis treatment's duration by supplementation of* Channa striata*. The result of study is useful to the government to create appropriate policy related to accelerate the treatment of tuberculosis in Jember regency and Situbondo Regency, especially in East Java Province-Indonesia.

## 2. Materials and Methods

### 2.1. Study Area and Time

The study was conducted in work area of primary health care in Jember Regency and Situbondo Regency. Jember and Situbondo had 49 units and 17 units of primary health care, respectively. The study was held in May-December 2017. The sputum test of tuberculosis patient was examined in Jember Chest Hospital (Rumah Sakit Paru Jember), in which Jember Chest Hospital was the only government hospital in 7 regencies (Eastern Pasuruan, Probolinggo, Lumajang, Jember, Situbondo, Bondowoso, and Banyuwangi), East Java-Indonesia, where the service focused on lung disease, particularly tuberculosis.

### 2.2. Method and Study Design

**Figure 1:**
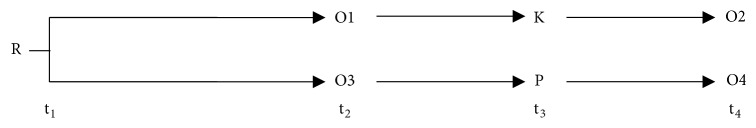
The study design of* Channa striata* and the acceleration of tuberculosis treatment. Description: R: randomize, K: control group (tuberculosis patient without treatment), P: intervention group (tuberculosis patient with treatment), O1,2: observational of control group, and O3,4: observational of intervention group.

The study was truly experimental, in which all of influential variables except intervention can be controlled. The author used true experiment to get the valid result, and the intervention of the study can be managed randomly. The treatment of the study was by supplementation of* Channa striata *(ikan gabus) extract to respondent (intervention or treatment group), in which control group was administered placebo. In addition, the respondent's sputum was examined in Jember Chest Hospital to recognize the availability of* Mycobacterium tuberculosis* each week within a month. The design of the study used randomized pretest-posttest only control group design that was showed by Figure 1. The randomized pretest-posttest only control group: the authors used randomization to determine the sample group of the study, where control group was used as comparator with intervention group. In addition, the author also examined the effect of* Channa striata* supplementation before and after administered to respondent.

The study was conducted on an ambulatory basis, where all respondents performed antibiotic treatment for tuberculosis in their house. The procedure of this study referred to the policy of Health Ministry of Indonesia, in which all new tuberculosis patients must get 6-month-full antibiotic treatment for tuberculosis in Indonesia. The medication for tuberculosis patient only with* Channa striata* is not appropriate in Indonesia, where there is no policy that regulated this issue. Therefore, this study used the policy of Health Ministry of Indonesia related to tuberculosis diagnosis and treatment as guidelines, in which the new tuberculosis patient must get standard antibiotic treatment for tuberculosis.

The prescription of tuberculosis antibiotic treatment was classified into two categories, namely, (a) primary drug (isoniazid, rifampicin, ethambutol, streptomycin, and pyrazinamide) and (b) secondary drug (exyonamid, para aminosalicylate, cycloserine, amikacin, capreomycin, and kanamycin). The function of* Channa striata* extract was as complementary supplement to accelerate tuberculosis treatment by increasing the nutritional status, and the* Channa striata* extract of this study is permitted to consume by Health Ministry of Indonesia (Registered Number: P-IRT: 202350901620).

### 2.3. Population and Sampling

The population of the study was all of new positive pulmonary tuberculosis patient that performed standard antibiotic treatment for tuberculosis in primary health care of Jember and Situbondo Regency, and respondent agreed to participate in research voluntarily. The total of pulmonary tuberculosis patients was 2,733 cases, in which the distribution of patients in Jember and Situbondo was 2,176 patients and 557 patients, respectively. Based on Kelsey, the total samples of control and intervention group are 100 respondents, respectively (Confidence Interval: 95%; Power: 90%; Ratio 1:1); therefore the total sample of the study was 200 respondents. In addition, the proportionated to size method was used to distribute the sample. The total samples of control and intervention group in the study is 97 respondents and 103 respondents, respectively.

There were exclusion criteria in this study, where the exclusion criteria were used to control the potential confounding variables, such as tuberculosis similar disease. Therefore, the authors can ensure the positive effect of* Channa striata* supplementation within the acceleration of tuberculosis treatment by shortening the tuberculosis duration recovery. The exclusion criteria of this study are (a) HIV/AIDS patients, (b) diabetes mellitus patients, (c) MDR patients, (d) respondent who did not take* Channa striata* supplement or placebo regularly (3x1 day), and (e) tuberculosis patients who fail drug treatment. Those respondents will be drop out of study or lost to follow-up.

### 2.4. Channa striata Supplementation

All respondents of this study were divided into 2 group by randomization, namely, intervention group and control group, where all respondents did not know their status in the group (blinding process). The intervention group performed not only standard antibiotic treatment for tuberculosis but also* Channa striata* supplementation. Meanwhile, the control group performed standard antibiotic treatment for tuberculosis and placebo supplementation. The tuberculosis antibiotic drug,* Channa striata* supplement, and placebo were administered to respondent by nurse in primary health care, in which the supplementation of* Channa striata* was 3 times a day during a month.

The 500 mg of extract or supplement of* Channa striata* that was registered in Health Ministry of Indonesia was administered to intervention group, in which the* Channa striata* supplement contains 90%* Channa striata* extract and 10% others. In addition, the supplement of* Channa striata* had several nutrients such as protein (80.9%), albumin (12.5%), and polyphenol bioflavonoid (6.6%). During study, the authors also used field research assistant to supervise and to monitor antibiotic drug-,* Channa striata*-, and placebo-used among respondent daily, where the respondent will be a dropout if they did not take standard tuberculosis antibiotic treatment and* Channa striata* extract or placebo regularly. In addition, the field research assistant also asked the respondent about the effect or complaint after taking* Channa striata*, in which respondent who got negative effect after administered with* Channa striata* will be referred to hospital under specialist doctor's control.

### 2.5. Collection, Handling, and Microscopic Examination of Sputum

The process of collection, handling, and microscopic examination of sputum was conducted by trained staffs, where the collection and handling process was performed by nurse in primary health care and the microscopic examination was conducted by health staff in Jember Chest Hospital. The sputum of respondent was collected every week (week 0-4) in a month or Day-0 (week-0), Day-7 (week-1), Day-14 (week-2), Day-21 (week-3), and Day-28 (Week-4), in which respondent should check the sputum in health primary care in Jember and Situbondo. The sputum was collected and putted on safety container (cylinder-form container) and directly delivers to Jember Chest Hospital. Therefore, this procedure can prevent the sputum damaged. The steps of microscopic examination of respondent's sputum consist of sputum culture, sputum-coloured culture, and finally the microscopic examination of sputum's smear. The process of sputum-coloured culture was conducted based on ziehl-neelsen technique, and the authors used International Union against Tuberculosis and Lung Disease (IUATLD) guideline to determine the availability of acid fast bacilli on the smear with 1,000x magnitude of microscopic examination. In addition, the authors used the number of acid fast bacilli in smear as indicator of acceleration of tuberculosis treatment after administered by* Channa striata* supplement. If there is an acid fast bacilli (AFB) in the smear, the smear is positive* Mycobacterium tuberculosis*.

The indicator of* Mycobacterium tuberculosis* availability in this study consists of negative, positive 1, positive 2, positive 3, and positive >3. The value of positive* Mycobacterium tuberculosis* in sputum was based on 3 stage of sputum examination, namely, sputum examination during a visit, in morning, and sputum collection. If the AFB was found within 3 stages, it is classified as positive 3. Meanwhile, positives 2 and 1 mean AFB was found in 2 stages and 1 stage of sputum examination, respectively. In addition, the sputum was classified as negative if the AFB was not found in these 3 stages.

### 2.6. Data Analysis

The study used Chi-square to analyze the data in SPSS version 22. Chi-square was used to determine the effect of* Channa striata* supplementation toward the acceleration of tuberculosis treatment. The significance level of the study is 5% (*α*= 0.05), and the confidence level is 95%.

## 3. Results and Discussion

### 3.1. The Characteristic of Respondent

Figure 1 presents the characteristic of respondent of all groups. Based on Table 1, the proportion of male is higher than female with 52%, and 37.5% of respondent age is >50 years old. Furthermore, most of respondents have low education (elementary level) with 38%, in which 18.5% of respondent are not educated.

### 3.2. The Distribution of Tuberculosis Sputum Status

Based on Table 2, the proportion of respondent with positive 2 on intervention group is high with 37.9% in week 0, which is higher than positive 1 (26.2%), positive 3 (26%), and negative (10.7%) category. Furthermore, the proportion of respondent with positive 3 on control group is high with 34%, which is higher than positive 2, positive 1, and negative category with 30.9%, 20.6%, and 13.4%, respectively.

Based on statistical test, the P value is 0.401 (P> *α*; *α*=0.05). H0 is accepted, which means that there are no differences between intervention and control group related to sputum test result among tuberculosis patient at week 0.

### 3.3. Channa striata and the Acceleration of Tuberculosis Treatment

Based on Table 3, the proportion of respondent of both negative and positive 1 category on intervention group is higher than other categories with 35.9%. In control group, the proportion of respondent classified in positive 1 (29.9%) is higher than positive 2 (27.8%), positive 3 (16.5%), and negative category (23.7%).

Based on statistical test, the P value is 0.045 (P< *α*; *α*=0.05). Therefore, H0 is rejected, which means that there are differences between* Channa striata *supplementation and the acceleration of tuberculosis treatment among patients at week 1.

Based on Table 4, the proportion of respondent of negative category in intervention group is higher than other categories with 56.3%. In control group, the proportion of respondent classified in positive 1 is high with 39.2%. It is higher than negative category (37.1%).

Based on statistical test, the P value is 0.086 (P > *α*; *α*=0.05). Therefore, H0 is accepted, which means that there are no differences between* Channa striata *supplementation and the acceleration of tuberculosis treatment among patients at week 2.

Based on Table 5, the proportion of respondent classified in negative category in intervention group is higher than other categories with 70.9%. Furthermore, the proportion of respondent of negative category in control group is high with 49.5%. It is higher than positive 1, positive 2, and positive 3 with 32%, 9.3%, and 7.2%, respectively.

Based on statistical test, the P value is 0.019 (P < *α*; *α*=0.05). Therefore, H0 is rejected, which means that there are differences between* Channa striata *supplementation and the acceleration of tuberculosis treatment among patients at week 3.

Based on Table 6, the proportion of respondent classified in negative category in intervention group is the highest with 90.3%. Furthermore, the proportion of respondent of negative category in control group is high with 68%. It is higher than positive 1, positive 2, and positive 3 with 21.6%, 6.2%, and 2.1%, respectively.

Based on statistical test, the P value is 0.005 (P < *α*; *α*=0.05). Therefore, Ho is rejected, which means that there are differences between* Channa striata *supplementation and the acceleration of tuberculosis treatment among patients at week 4.

Tuberculosis (TB) is communicable disease caused by bacteria called* Mycobacterium tuberculosis, *in which the bacteria usually attack not only the lung but also any part of the body such as the kidney, spine, brain [17], nerve, circulation, skeleton, and joint [11].* M. tuberculosis* has square shape, which is classified in gram-positive basil. The bacteria are easy to disappear after contact with sunlight directly [6]. Tuberculosis is classified as chronic disease, and the bacteria are spread by air [18]. Based on socioeconomic aspect, the transmission of tuberculosis is also affected by urbanization, crowded area, and poverty [19]. Tuberculosis is one of public health problems in worldwide especially in developing countries [2] that has high level of morbidity and mortality of tuberculosis [18]. The major incident of tuberculosis (85%) in worldwide occurred in Asia and Africa [20].

The sputum of tuberculosis respondent is collected periodically by primary health officer of Jember and Situbondo on week 0, week 1, week 2, week 3, and week 4, in which the sputum is examined in Jember Chest Hospital. Jember Chest Hospital (Rumah Sakit Paru Jember) is one of chest-concerned hospitals in East Java Province that the work area of the hospital is Eastern Pasuruan, Probolinggo, Lumajang, Jember, Situbondo, Bondowoso, and Banyuwangi—Indonesia. In addition, the sputum is examined to determine the level of the tuberculosis.

The distribution of tuberculosis level of intervention and control group without* Channa striata *supplementation is shown by Table 2, in which most of respondents of intervention and control group are classified in positive 2 (37.9%) and positive 4 (34%), respectively. Besides, based on Table 2, the proportion of respondent with negative category in intervention and control group is 10.7% and 13.4%, respectively, with the result that respondent in control group that has negative status of tuberculosis is higher than intervention group. Furthermore, based on Chi-square test, the P value is 0.401 (P> *α*; *α*=0.05). It shows that there are no differences between intervention and control group related to sputum test result of tuberculosis patient at week 0.

The distribution of tuberculosis level subsequent to* Channa striata *supplementation of intervention and control group is shown by Tables 3–6, in which the sputum was collected and examined in week 1, week 2, week 3, and week 4. Table 3 showed the distribution of tuberculosis sputum test between intervention and control group in week 1. Based on Table 3, respondent that is classified in negative category of tuberculosis level in intervention group is high with 35.9%. It is higher than the proportion of respondent with negative category in control group (23.7%). Furthermore, the distribution of tuberculosis sputum test between intervention and control group in week 2 is shown by Table 4. Respondent that is classified in negative category of tuberculosis level in intervention group is high with 56.3%. It is higher than the proportion of respondent with negative category in control group (37.1%). Table 5 showed the distribution of tuberculosis sputum test between intervention and control group in week 3. Respondent that is classified in negative category of tuberculosis level in intervention group is high with 70.9%. It is higher than the proportion of respondent with negative category in control group (49.5%). Table 6 showed the distribution of tuberculosis sputum test between intervention and control group in week 4. Respondent that is classified in negative category of tuberculosis level in intervention group is high with 90.3%. It is higher than the proportion of respondent with negative category in control group (68%).

Generally,* Channa striata* supplementation in this study is significantly related to the acceleration of tuberculosis recovery. It is caused by the elevating distribution of respondent classified in negative category of tuberculosis in intervention group on week 1, week 2, week 3, and week 4 with 35.9%, 56.3%, 70.9%, and 90.3%, respectively. Furthermore, based on statistical test, the P value of* Channa striata *supplementation toward the acceleration of tuberculosis treatment week 1, week 3, and week 4 is 0.045, 0.019, and 0.005 (P< *α*; *α*=0.05), respectively. Therefore, there are differences between* Channa striata *supplementation and the acceleration of tuberculosis treatment among respondent.

*Channa striata* is cheap resource in Thailand [21], which is cultivated restrictedly in Thailand and Indonesia [22].* Channa striata* is vulnerable to aquatic Mycobacterium [21, 22]. However,* Mycobacterium tuberculosis* is not founded in* Channa striata* [23]. The extract of* Channa striata *through chloroform solvent contains several amino acids (aspartate acid, glutamate acid, serine, glycine, histidine, arginine, threonine, alanine, proline, tyrosine, valine, methionine, leucine, phenylalanine, and lysine) and fatty acid (myristic acid, palmitate acid, stearate acid, heptadecanoic acid, palmitoleic acid, oleate acid, linoleic acid, and arachidonic acid) [14]. The major amino acid found in* Channa striata* extract is glycine (35.77% of protein total) and alanine (10.19% of protein total). Besides, the major fatty acid found in* Channa striata* extract is palmotoleic acid (35.93% of fatty acid total), oleate acid (22.96% of fatty acid total), stearate acid (15.31% of fatty acid total), and linoleate acid (11.45% of fatty acid total) [14]. Based on pharmacology activities, the aqueous extract of* Channa striata* on male mice (25-30 g) possessed a concentration-dependent antinociceptive activity [24].

The extract of* Channa striata* was administered orally to osteoarthritis-induced rabbits (OA), in which the result is that there was a significant improvement in the density of Protein Gene Product (PGP) of 9,5-immunoreactive nerve fibers in the synovial membrane of treated animals [25]. Furthermore, the extract of* Channa striata* possesses antifungal activities in restricted spectrum [26]; however the acid extract of mucus has bactericidal activity that reduced the bacteria pathogen growth among human, such as* Klebsiella pneumoniae, Pseudomonas aeruginosa, *and* Bacillus subtilis *[27]. The cream formulation of* Channa striata* extract can heal the wound in Sprague-Dawley rats (250-300 g) [28]. Besides, the* Channa striata* is also formulated in spray form to heal the wound [29, 30]. The aerosol form is created from butane, propene, and the combination of butane-propene as propellant [30]. Furthermore, Maj Jais [31] noted that the extract of* Channa striata *without bone is unable to decrease the blood sugar and HDL (High Density Lipoprotein) level toward Sprague-Dawley rat and mice.

The limitation of study is that the authors cannot provide the information about the effect of* Channa striata* among multidrug-resistant (MDR) tuberculosis patients. Therefore, future research is needed to find the effect of* Channa striata *supplementation with standard antibiotic for MDR patients related to accelerating the MDR TB recovery.

## 4. Conclusions

Based on the result of the study, the proportion of respondent classified in negative category in intervention group within week 0, week 1, week 2, week 3, and week 4 is 10.7%, 35.9%, 56.3%, 70.9%, and 90.3%, respectively. Besides, the proportion of respondent classified in negative category in control group within week 0, week 1, week 2, week 3, and week 4 is 13.4%, 23.7%, 37.1%, 49.5%, and 68%, respectively. Furthermore, based on Chi-square* test, *the P value of* Channa striata *supplementation toward the acceleration of tuberculosis treatment week 1, week 3, and week 4 is 0.045, 0.019, and 0.005 (P< *α*; *α*=0.05), respectively. Therefore, there are differences between* Channa striata *supplementation and the acceleration of tuberculosis treatment among respondent, in which* Channa striata* treatment in this study is significantly related to the acceleration of tuberculosis recovery.

## Figures and Tables

**Table 1:** The respondent characteristics.

	Respondent Characteristic		
	Categories	Number	Percentage (%)
Sex	Male	104	52.0
	Female	96	48.0

Age	<20	16	8
	20-29	37	18.5
	30-39	29	14.5
	40-49	43	21.5
	>50	75	37.5

Education	None	37	18.5
	Elementary	76	38
	Junior High School	37	18.5
	Senior High School	47	23.5
	University	3	1.5

**Table 2:** The distribution of tuberculosis sputum status at week 0.

Categories	Negative (%)	Positive 1 (%)	Positive 2 (%)	Positive 3 (%)	Positive >3 (%)	Total
Intervention	10.7	26.2	37.9	26	0	100
Control	13.4	20.6	30.9	34	1	100

Total	12	23.5	34.5	29.5	0.5	100

**Table 3:** The sputum test of tuberculosis patients at week 1.

Categories	Negative (%)	Positive 1 (%)	Positive 2 (%)	Positive 3 (%)	Positive >3 (%)	Total
Intervention	35.9	35.9	21.4	4.9	2	100
Control	23.7	29.9	27.8	16.5	2	100

Total	30	33	24.5	10.5	2	100

**Table 4:** The sputum test of tuberculosis patients at week 2.

Categories	Negative (%)	Positive 1 (%)	Positive 2 (%)	Positive 3 (%)	Positive >3 (%)	Total
Intervention	56.3	30.1	10.7	2.9	0	100
Control	37.1	39.2	14.4	7	1	100

Total	47	34.5	12.5	5	0.5	100

**Table 5:** The sputum test of tuberculosis patients at week 3.

Categories	Negative (%)	Positive 1 (%)	Positive 2 (%)	Positive 3 (%)	Positive >3 (%)	Total
Intervention	70.9	21.4	4.9	1	1.9	100
Control	49.5	32	9.3	7.2	2	100

Total	60.5	26.5	7	4	2	100

**Table 6:** The Sputum test of tuberculosis patients at week 4.

Categories	Negative (%)	Positive 1 (%)	Positive 2 (%)	Positive 3 (%)	Positive >3 (%)	Total
Intervention	90.3	8.7	1	1	0	100
Control	68	21.6	6.2	2.1	2	100

Total	77	17	3.5	1.5	1	100

## Data Availability

The data used to support the findings of this study are available from the first author and corresponding author upon request.
